# The Alteration Profiles of m^6^A-Tagged circRNAs in the Peri-Infarct Cortex After Cerebral Ischemia in Mice

**DOI:** 10.3389/fnins.2022.869081

**Published:** 2022-06-07

**Authors:** Yudi Li, Hanzhao Li, Yang Luo, Xiaoqiang Li, Zhefeng Chen, Wanzhou Zhang, Fangming Li, Li Ling

**Affiliations:** ^1^Department of Neurology, Shenzhen Hospital, Southern Medical University, Shenzhen, China; ^2^School of Physical Education, Southwest University, Chongqing, China; ^3^Department of Neurology, Affiliated Xiaolan Hospital, Southern Medical University, Xiaolan People's Hospital, Zhongshan, China; ^4^Department of Neurology, Shenzhen University General Hospital, Shenzhen University Clinical Medical Academy, Shenzhen, China

**Keywords:** circRNA, N^6^-methyladenosine (m^6^A), m^6^A-related methyltransferases, middle cerebral artery occlusion, cerebral infarction

## Abstract

The N6-methyladenosine (m^6^A) modification acts as a dynamic regulatory factor in diseases by regulating the metabolism and function of the transcriptome, especially mRNAs. However, little is known regarding the functional effects of m^6^A modifications on circRNAs. In this research, we established a distal middle cerebral artery occlusion (MCAO) model in adult C57BL/6J mice. The mice were divided into three groups: sham surgery, 3 days after MCAO (3d), and 7 days after MCAO (7d). Reverse transcription quantitative polymerase chain reaction (RT-qPCR) demonstrated that the mRNA expression levels of m^6^A-related methyltransferases (METTL3, METTL14), demethylases (FTO, ALKBH5), and reading proteins (YTHDF1, YTHDF3) altered compared to the sham group. Furthermore, the translation level of ALKBH5 and YTHDF3 was significantly decreased in the 3d group while increased in 7d group. Methylated RNA immunoprecipitation (MeRIP) and circRNA microarray indicated 85 hypermethylated and 1621 hypomethylated circRNAs in the 3d group. In the 7d group, the methylation level increased in 57 and decreased in 66 circRNAs. Subsequently, our results were verified by MeRIP-qPCR. Bioinformatics analysis was performed to analyze the functions of differentially m^6^A-modified circRNAs. We found some m^6^A modified-circRNAs associated with cerebral infarction, providing a new direction for the molecular mechanism of stroke.

## Introduction

Cerebral infarction causes transcriptional and post-transcriptional alterations including RNA expression levels and epigenetic modifications, resulting in a series of complicated post-stroke pathophysiological processes, such as glutamate excitotoxicity, oxidative stress, apoptosis, nerve regeneration, and angiogenesis (Mehta et al., 2007).

Studies of RNA modification are known as epigenetic transcriptomics. Recent studies have indicated that epigenetic changes occur during the pathophysiological course of diverse diseases as regulators of RNAs (Niu et al., 2013). At present, more than 170 classes of RNA modifications have been identified, among which N6-methyladenosine (m^6^A) is the most abundant and common modification in Eukaryotes, particularly in the mammalian brains (Dominissini et al., 2012). The m^6^A modification refers to the methylation of the sixth nitrogen atom in adenosine, which is catalyzed by methyltransferase complexes, such as methyltransferase-like (METTL) 3 and 14 (METTL3 and METTL14), and Wilms' tumor 1-associated protein (WTAP); eliminated by demethylases, such as fat mass and obesity-associated protein (FTO) and alkylation repair homolog protein 5 (ALKBH5); and recognized by m^6^A readers, such as YTH domain family (YTHDF) 1, 2, and 3 (YTHDF1, YTHDF2, and YTHDF3) (Shi et al., 2019). Thus, the m^6^A modification is a dynamic and reversible process. The majority of m^6^A modifications occur near the 3′UTR and the mRNA stop codon, and regulate various molecular functions in mRNAs related to transcription, alternative splicing, nuclear export, translation, stability, and degradation (Niu et al., 2013). It has been researched that m^6^A-related enzymes play a critical role in ischemic diseases. FTO has been reported to promote angiogenesis and reduce myocardial fibrosis after myocardial ischemia, however, in the brain, it is predominantly expressed in neurons, whereas downregulation of FTO and ALKBH5 after stroke can induce secondary brain injury by destroying neurons (Chokkalla et al., 2019; Mathiyalagan et al., 2019; Xu et al., 2020). METTL3, METTL14, and YTHDF1 have been shown to regulate axon regeneration, neuronal apoptosis, neurogenesis, and inflammation after cerebral ischemia (Weng et al., 2018; Si et al., 2020; Zhang et al., 2020), which indicates that the m^6^A modification may be involved in a series of post-stroke molecular events.

Non-coding RNAs, especially circRNAs, show tissue specificity during development and are highly expressed in the brain (Rybak-Wolf et al., 2015). Studies have shown that circRNAs are specifically enriched in the subcellular compartments of neurons, including dendrites, axons, and synapses (Rybak-Wolf et al., 2015; You et al., 2015; Shigeoka et al., 2016). Therefore, tracking changes in circRNAs and their m^6^A modification levels may provide new insights into the pathogenesis of cerebral ischemia from the perspective of epigenetics, thereby improving intervention and prognosis. CircRNAs can regulate secondary brain injury and repair by binding with proteins and acting as “sponges” for miRNA (Wu et al., 2019; Yang et al., 2020). However, the upstream regulatory mechanism for circRNAs remains unknown. Recently, a study focused on the differences in m^6^A modifications of mRNAs lncRNAs after transient cerebral ischemia (Chokkalla et al., 2019), but the m^6^A modification of circRNA is not yet known. Therefore, in this study, we screened the entire genome for m^6^A-modified circRNAs around the site of cerebral infarction to explore the potential significance of m^6^A-modified circRNAs in stroke.

## Methods and Materials

### Cerebral Infarction in Mice

All experimental procedures were approved by the Institutional Animal Use Committee of the Shenzhen Hospital of Southern Medical University. Animals were raised in a SPF room with free access to food and water, under a controlled temperature (25 ± 2°C) and a 12/12 h day/night cycle The surgery was performed in accordance with the guidelines for the care and use of laboratory animals. C57BL/6J mice weighing 23–26 g were randomized to three groups: the 3d after MCAO, 7d after MCAO, and sham groups. After anesthetizing the mice with isoflurane, we made a 1 cm incision between the left ear and eye, dissected the temporal muscle, and exposed the left side of the skull under an operating microscope. Next, a small hole was drilled, and a distal middle cerebral artery occlusion (MCAO) was performed with a bipolar electrosurgical knife, leading to a permanent infarction of the local cerebral cortex. And then the bilateral common carotid arteries were immediately occluded for 7 minutes. The sham group underwent the same surgical procedure without the MCAO. During surgery and recovery, the body temperature of the mice were maintained at 37°C.

### TTC Staining

Three mice were used for histological staining at 24 h after MCAO. Briefly, the mice were killed by cervical dislocation, and brain tissues were isolated and frozen at −20°C in refrigerator for 5 mins. Coronal sections were sliced into 1 mm and maintained at 37°C in 2% 2,3,5-triphenyltetrazolium chloride (TTC) solution (Sigma Aldrich, St. Louis, MO, USA) for 30 min. TTC staining can effectively detect the location of the primary cerebral infarction after MCAO.

### H&E Staining

One week after cerebral infarction operation, the brain tissue was collected and fixed with 4% paraformaldehyde for 1 week, and then embedded in paraffin. Following alcohol dehydration and xylene dewaxing, coronal sections were stained with hematoxylin for 3 min, washed, and then eosin for 2 min. Finally, sections were sealed with neutral gum after being dehydrated.

### m^6^A Immunoprecipitation (MeRIP)

Total RNA (1–3 μg) and m^6^A spike-in control mixture were added to immunoprecipitation (IP) buffer (50 mmol/L Tris-HCl, pH 7.4; 150 mmol/L NaCl; 0.1% NP40; and 40 U/μL RNase inhibitor), incubated with 2 μg anti-m^6^A rabbit polyclonal antibody (Synaptic Systems, 202003) at 4°C for 2 h, washed, resuspended in 20 μL Dynabeads™ M-280 sheep anti-rabbit IgG suspension (Invitrogen, 11203D), and incubated at 4°C for 2 h. The beads were then washed three times with 500 μL of IP buffer and twice with 500 μL of wash buffer [50 mmol/L Tris-HCl, pH 7.4; 50 mmol/L NaCl; 0.1% NP 40; and 40 U/μL RNase inhibitor (Enzymatics, Y9240L)]. The enriched RNA was eluted with 200 μL elution buffer (10 mmol/L Tris-HCl, pH 7.4; 1 mmol/L EDTA; 0.05% SDS; and 40 U proteinase K) and extracted using acid phenol-chloroform and ethanol precipitation. MeRIP assays were performed in three replicates in each group (*n* = 3).

### Microarray Hybridization of Methylated CircRNAs

The immunoprecipitate was digested with RNase R to remove linear RNA, amplified, and labeled with super RNA Labeling Kit (Cy3 [for “sup”, supernatant] and Cy5 [for “IP”]) in accordance with the manufacturer's instructions, and then purified using the RNeasy Mini Kit. After fragmentation, the circRNAs were hybridized to an m^6^A-circRNA epi-transcriptomic microarray slide (Arraystar) and incubated at 65°C for 17 h in a hybridization oven (Agilent, Santa Clara, CA, USA). The hybridized arrays were washed, fixed, and scanned using an Agilent Scanner G2505C.

### Analysis of Microarray Data

Acquired array images were analyzed using Agilent Feature Extraction software (version 11.0.1.1). Raw intensities of IP (immunoprecipitated, Cy5-labeled) were normalized at a criterion of log2-scaled intensities in spike-in RNA. The percentage of the IP (Cy5-labeled) normalized intensities was considered as m^6^A methylation levels. Differentially m^6^A-modified circRNAs were identified between the two comparison groups by filtering with the fold change and statistical significance (*P*-value) thresholds. Expression of circRNAs were calculated by the normalized signal value of “IP” plus “sup”, the differentially expressed circRNAs were screened at the criterion of with fold change >1.5 and *P* < 0.05.

### Reverse Transcription Quantitative Polymerase Chain Reaction

After collecting samples at 3d and 7d after MCAO, 1 μg of total RNA was reverse transcribed to cDNA. RT-qPCR was performed to determine the mRNA levels of m^6^A-related enzymes (METTL3, METTL14, FTO, WTAP, ALKBH5, YTHDF1, and YTHDF3) by the Applied Biosystems 7300/7500 Real-Time PCR System (Foster City, CA, USA). Additionally, to further confirm the results from the m^6^A immunoprecipitation and microarray, we randomly selected four differentially m^6^A-modified circRNAs (mmu_circRNA_27268, mmu_circRNA_20673, mmu_circRNA_32905, and mmu_circRNA_29864) in samples of 3d after MCAO and detected their level by MeRIP-qPCR analysis. In brief, equal quantity of immunoprecipitated RNA samples were reverse-transcribed to cDNA using SuperScriptTM III Reverse Transcriptase (Invitrogen, 18080-044), and amplified with gene-specific primers for the circRNAs (Table 1). PCR assays were performed in six replicates in each group (*n* = 6). All results were analyzed by 2^ΔΔCT^ method.

**Table 1:** The primer sequence of the four differentially m^6^A-modified circRNAs and m^6^A-related enzymes.

	**Bidirectional primer sequence**
mmu_circRNA_27268	F:5′ CTTTGGTCAAGGAGGCAGAT3′ R:5′ TTTGACAACCGACTGTACCTATTA3′
mmu_circRNA_29864	F:5′ GACTCCTTTTTGCGGCTTG3′ R:5′GTGGAGGGCATTTGGAACAT3 ′
mmu_circRNA_32905	F:5′CATTGTCTAAAGATGCTTCTGTCAG3 ′ R:5′GTGACCCAGGACAACCAAAAG3 ′
mmu_circRNA_20673	F:5′ AACCAAAGAGTTCAGACGTGAGA3′ R:5′CGTTTTTCTGAGGATGGTGTTAC3 ′
METTL3	F:5′GGGCACTTGGATTTAAGGAACC3′ R:5′CTTAGGGCCGCTAGAGGTAGG3 ′
METTL14	F:5′GAGCTGAGAGTGCGGATAGC3′ R:5′GCAGATGTATCATAGGAAGCCC3′
FTO	F:5′TTCATGCTGGATGACCTCAATG3′ R:5′GCCAACTGACAGCGTTCTAAG3′
WTAP	F:5′ATGGCACGGGATGAGTTAATTC3′ R:5′TTCCCTTAAACCAGTCACATCG3′
ALKBH5	F:5′GCATACGGCCTCAGGACATTA3′ R:5′TTCCAATCGCGGTGCATCTAA3′
YTHDF1	F:5′CACAGTGACTCCCTCAACAAG3′ R:5′AGGTGGTAACATCCCCAATCTT3′C
YTHDF3	F:5′AACCAGGGGCATTAGGAAATACC3′ R:5′TCCACTTGTTCCCCATGTAGAG3′

### Western Blots Analysis

Around 50 μg of protein samples extract was resolved in 10% sodium dodecylsulfate-polyacrylamide gel electrophoresis (SDS-PAGE) after denaturation and transferred to PVDF membranes. The membranes were blocked in 5% non-fat milk in tris-buffered saline solution (TBS) for 1.5 h at room temperature and then probed with the primary antibodies: rabbit anti-ALKBH5 (Abcam, ab195377, 1:1,000) and rabbit anti-YTHDF3 (Abcam, ab220161, 1:1,000). After overnight incubation at 4°C, the membranes were washed three times with TBS-T and incubated for 1 h at room temperature with an appropriate secondary antibody: horseradish peroxidase-labeled anti-rabbit IgG (1:5,000). The protein bands were visualized using the enhanced chemiluminescence method, and the intensity of band was quantified using Image J software (Version 1.51j8, NIH, Bethesda, MD, USA).

### Function and Pathways Analyses

The functions of differentially m^6^A-modified circRNAs' parent genes and three verified ones' target genes were analyzed by Gene Ontology (GO) analysis (http://www.geneontology.org), and their significant pathways were explored by the Kyoto Encyclopedia of Genes and Genomes (KEGG) pathway analyses (http://www.genome.jp/kegg/) using the DAVID bioinformatics database (https://david.ncifcrf.gov).

### Prediction of Network of CircRNA-miRNA-mRNA

Three verified m^6^A-circRNAs were used to predicted miRNAs by Arraystar's homemade software based on TargetScan and miRanda, and top ten possible miRNAs were screened. Additionally, mRNAs of these ten miRNAs were predicted by miRanda. The **network of circRNA-miRNA-mRNA** was visualized by Cytoscape.

### Statistical Analysis

Our results were analyzed using Statistical Program for Social Sciences (SPSS) software (version 22.0; SPSS, Chicago, IL, USA) and are presented as means ± standard deviations. The student *t* test was used to detect the significant differences between two groups, one-way ANOVA was used for comparisons between multiple groups, and *P* < 0.05 was taken as statistically significant.

## Results

### Establishment and Validation of the Cerebral Infarction Model

**Figure 1:**
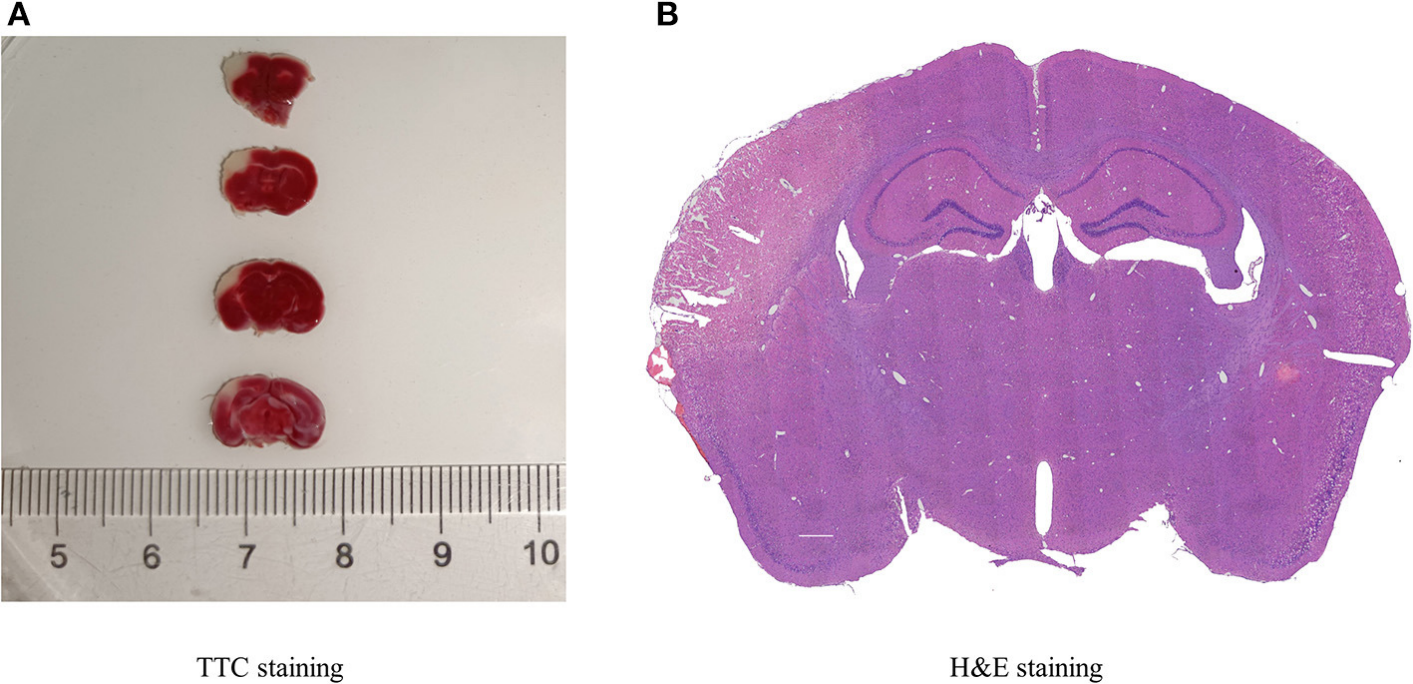
Histopathology of cerebral infarction. **(A)** TTC staining displayed the infarcted areas in white, and the normal areas in red, (*n* = 3). **(B)** H&E staining presented the destruction of morphology in the infarct lateral cortex compared with the Contralateral cortex (*n* = 3).

TTC and H&E staining were used to confirm the infarction region of the brain after MCAO in mice (Figures 1A,B). TTC staining showed that the infarcted brain tissue was white, and the non-infarct region was red. Additionally, with H&E staining, the infarct area was pale, and the number of neurons was decreased, whereas the opposite was observed in normal tissue.

### The Transcriptional Levels of Methylation-Related Enzymes After Stroke in Mice

**Figure 2:**
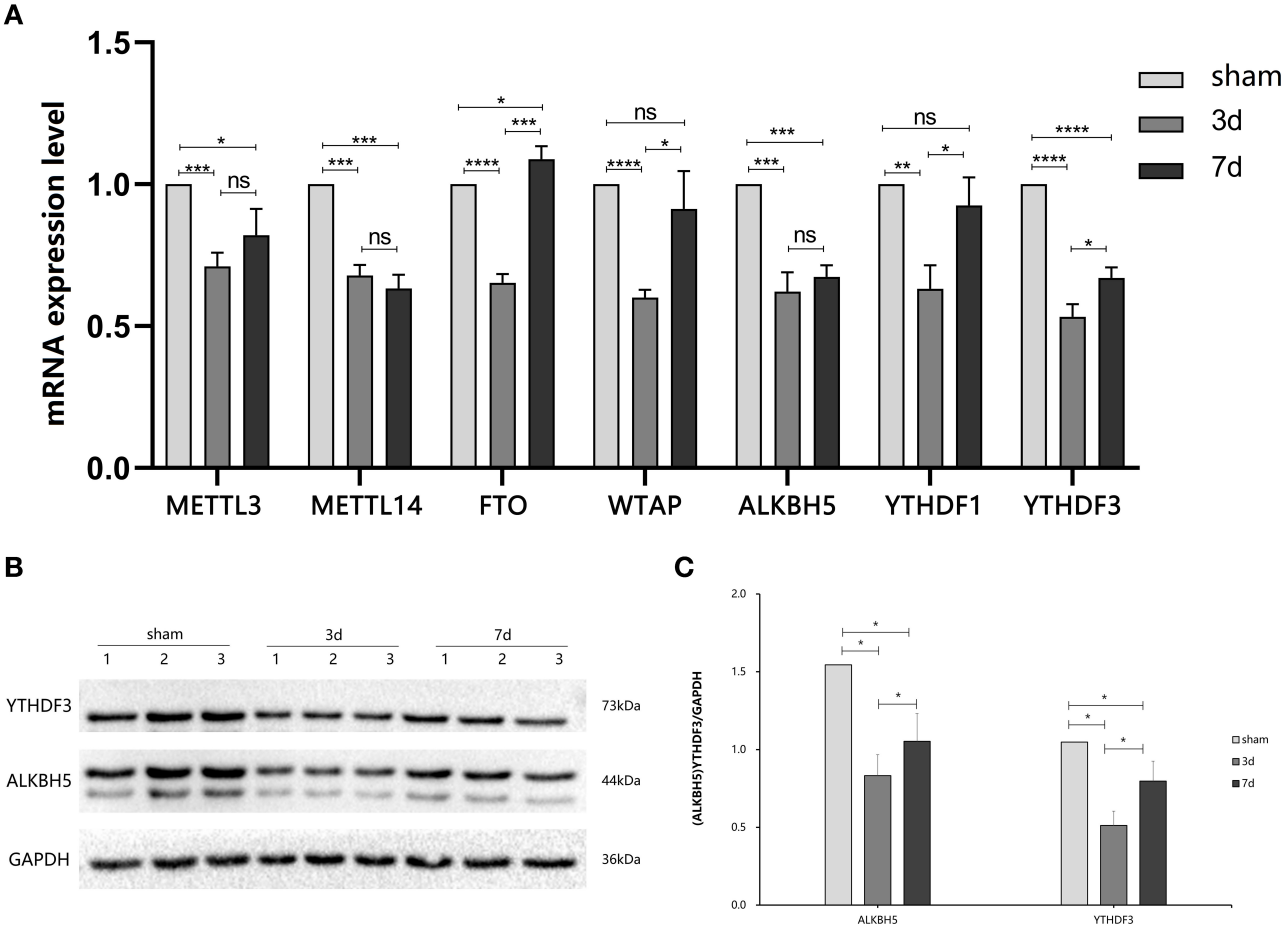
Expression of m^6^A related enzymes between MCAO and sham groups. **(A)** The mRNA expression level of m^6^A writers (METTL3, METTL14, and WTAP), m^6^A erasers (FTO, ALBKH5), and m^6^A readers (YTHDF1, YTHDF3) were changed at 3d or 7d after MCAO compared with the sham, (*n* = 6/group). **(B,C)** The protein YTHDF3 were significantly decreased in 3d group, while increased in 7d group, (*n* = 6/group). ^*^P < 0.05, ^**^P < 0.01, ^***^P < 0.001, ^****^P < 0.0001; ns, not significant.

To explore the changes in m^6^A methylation on circRNA transcripts, we first determined transcriptional levels of m^6^A-related enzymes in the peri-infarct region of the cerebral cerebral cortex (Figure 2A). We observed that mRNA expression of all enzymes tested was significantly downregulated at 3 days after MCAO compared with the sham group. At 7 days after MCAO, the transcriptional levels of METTL3, METTL4, ALKBH5, and YTHDF3 were decreased, whereas increased in FTO significantly. Interestingly, compared with the 3d group, except for METTL3, METTL14, and ALKBH5, significant increases in transcription of other genes occurred in the 7d group.

### ALKBH5 and YTHDF3 Showed Dynamic Alterations in Stroke

We further measured the translation level of m^6^A related enzymes in the area around cerebral cortex infarction (Figures 2B,C). We observed that ALKBH5 and YTHDF3 were significantly decreased at 3 and 7 days after MCAO compared with the sham group. Interestingly, ALKBH5 and YTHDF3 were increased at 7 days in comparison to 3 days after MCAO.

### Cerebral Infarction Changed the Level of m^6^A Modification and Expression Profile in CircRNA

**Figure 3:**
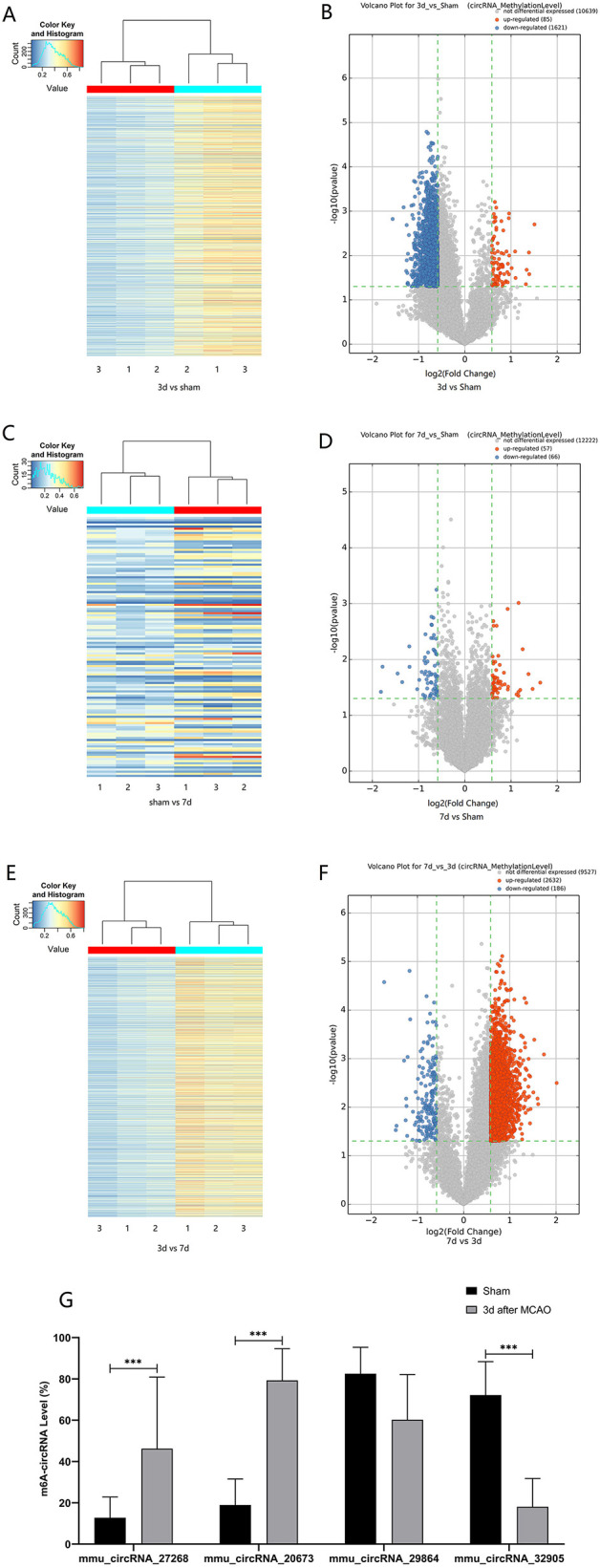
Alteration of m^6^A methylated-circRNAs. Different methylation modification in circRNAs in 3d **(A)** or 7d **(C)** vs. sham and 7d vs. 3d **(E)** are shown in heatmaps. The volcano plots display differentially m^6^A-methylated circRNAs in 3d **(B)** or 7d **(D)** vs. sham and 7d vs. 3d **(F)**: the green line means the screening criteria with fold change >1.5, red dots represent the hypermethylated circRNAs while green dots represent the hypomethylated ones. **(G)** The verified results of mmu_circRNA_27268, mmu_circRNA_20673, mmu_circRNA_32905, and mmu_ circRNA_29864 by MeRIP-qPCR in MCAO and sham groups (MeRIP assays, *n* = 3/group; MeRIP-qPCR, *n* = 6/group). ^*^P < 0.05, ^***^P < 0.001; ns, not significant.

The mRNA expression of m^6^A-related enzymes in the peri-infarct cortex was altered, suggesting that cerebral ischemia might change m^6^A modification. We speculated that the methylation modification of circRNAs around cerebral infarction was changed. We performed m^6^A immunoprecipitation and microarray analysis of circRNAs from cortex tissue surrounding the infarcted area after 3 and 7 days of permanent cerebral ischemia in mice. Heatmaps and volcano graphs showed differences in m^6^A methylation in circRNAs between the infarct groups and the sham group (fold change > 1.5, *P* < 0.05, *n* = 3/group) (Figures 3A–F). A total of 12,345 methylated circRNAs were found in our research. In comparison with the sham group, the m^6^A modification was significantly changed in 1,706 circRNAs, including 85 hypermethylated and 1,621 hypomethylated circRNAs at 3d after MCAO. However, the methylation levels increased in 57 circRNAs and decreased in 66 circRNAs at 7d after MCAO. In general, at 3d after operation, the hypomethylated transcripts reached to 95% in the m^6^A-circRNAs, and only 5% were hypermethylated, while the distribution of these two kinds of transcripts was nearly equal at 7d after MCAO compared to the sham group. Additionally, compared to the 3d group, 2632 circRNAs were hypermethylated, while 186 ones were hypomethylated in the 7d group. The top 10 hyper and hypo-methylated circRNAs with different treatments were listed as Tables 2–4.

**Table 2:** The top 10 hyper and hypo-methylated circRNAs between 3d vs. sham.

**CircRNA**	**m^**6**^A**	**Fold change**	***P* value**	**Chrom**	**Type**	**Gene**
mmu_circRNA_27268	hyper	2.84197	0.0019873	chr14	exonic	Appl1
mmu_circRNA_009696	hyper	2.61714	0.0085627	chr19	intronic	Malat1
mmu_circRNA_017958	hyper	2.50106	0.0443987	chr10	exonic	Anks1b
mmu_circRNA_000807	hyper	2.14106	0.0320552	chr17	sense overlapping	Rn45s
mmu_circRNA_20066	hyper	2.12641	0.0081174	chr1	sense overlapping	DQ715306
mmu_circRNA_005252	hyper	2.01853	0.019242	chr16	intronic	Cmss1
mmu_circRNA_19450	hyper	1.94602	0.0100234	chr7	sense overlapping	Calm3
mmu_circRNA_43940	hyper	1.94234	0.0011256	chr9	exonic	Arhgap32
mmu_circRNA_007113	hyper	1.94061	0.0265738	chr1	exonic	Rims1
mmu_circRNA_20673	hyper	1.9342	0.0014401	chr1	exonic	Clasp1
mmu_circRNA_24601	hypo	2.9504766	0.0015035	chr12	exonic	Itsn2
mmu_circRNA_38065	hypo	2.5043721	0.0008175	chr5	exonic	Cfap69
mmu_circRNA_44763	hypo	2.4079717	0.0223753	chr9	exonic	Dopey1
mmu_circRNA_38624	hypo	2.391517	0.0020564	chr5	exonic	Tbc1d19
mmu_circRNA_36570	hypo	2.3893449	0.0128245	chr4	exonic	Klhl32
mmu_circRNA_45982	hypo	2.3683147	0.0247834	chrX	exonic	Mid1
mmu_circRNA_45969	hypo	2.3645312	0.0431726	chrX	exonic	Ofd1
mmu_circRNA_39011	hypo	2.3522961	0.0412524	chr5	exonic	Bmp2k
mmu_circRNA_27887	hypo	2.3463249	0.0091444	chr14	exonic	Mtrf1
mmu_circRNA_19997	hypo	2.3328161	0.0451034	chr1	exonic	Mrps9

**Table 3:** The top10 hyper and hypo-methylated circRNAs between 7d vs. sham.

**CircRNA**	**m^**6**^A**	**Fold change**	***P* value**	**Chrom**	**Type**	**Gene**
mmu_circRNA_23150	hyper	3.1009042	0.0259945	chr11	exonic	Psme4
mmu_circRNA_38094	hyper	2.7648072	0.0337221	chr5	exonic	9330182L06Rik
mmu_circRNA_22281	hyper	2.5990145	0.0182551	chr10	exonic	Creb3l3
mmu_circRNA_25983	hyper	2.3819553	0.0065519	chr13	exonic	Larp4b
mmu_circRNA_28128	hyper	2.2970656	0.0351191	chr14	exonic	Itgbl1
mmu_circRNA_28040	hyper	2.2407646	0.0009723	chr14	exonic	Uggt2
mmu_circRNA_23280	hyper	2.2379241	0.0446396	chr11	exonic	Cnot6
mmu_circRNA_36627	hyper	2.2361166	0.0381938	chr4	exonic	Orc3
mmu_circRNA_24182	hyper	2.1735471	0.0422457	chr11	exonic	Acly
mmu_circRNA_38487	hyper	1.916812	0.0307721	chr5	exonic	Trmt44
mmu_circRNA_32468	hypo	3.5205166	0.03799	chr19	exonic	Pcgf5
mmu_circRNA_19150	hypo	3.4371052	0.0134725	chr15	sense overlapping	Arid2
mmu_circRNA_43936	hypo	2.740639	0.0178457	chr9	exonic	Prdm10
mmu_circRNA_19402	hypo	2.5589497	0.0254406	chr5	sense overlapping	Cdk8
mmu_circRNA_45325	hypo	2.2991652	0.0058466	chrX	exonic	Sytl5
mmu_circRNA_27622	hypo	2.2918234	0.0135477	chr14	exonic	Zdhhc20
mmu_circRNA_018245	hypo	2.0415875	0.0340724	chr13	exonic	Nln
mmu_circRNA_19824	hypo	1.981272	0.0231978	chr1	exonic	Adgrb3
mmu_circRNA_006216	hypo	1.9072746	0.0167186	chr12	exonic	Dtnb
mmu_circRNA_20158	hypo	1.8690753	0.0429307	chr1	exonic	Fam126b

**Table 4:** The top 10 hyper and hypo-methylated circRNAs between 7d vs. 3d.

**CircRNA**	**m^**6**^A**	**Fold change**	***P* value**	**Chrom**	**Type**	**Gene**
mmu_circRNA_28863	hyper	4.0392625	0.0031755	chr15	exonic	Srebf2
mmu_circRNA_38904	hyper	3.3380276	0.0008196	chr5	exonic	Uba6
mmu_circRNA_19765	hyper	3.0605701	0.0086643	chr1	exonic	Fam135a
mmu_circRNA_45965	hyper	3.0318816	0.0066543	chrX	exonic	Glra2
mmu_circRNA_33707	hyper	2.8852467	0.000408	chr2	exonic	Scn2a1
mmu_circRNA_42640	hyper	2.8462651	0.0049594	chr8	exonic	Kat6a
mmu_circRNA_30501	hyper	2.7141039	0.0023387	chr17	exonic	Ptchd4
mmu_circRNA_19249	hyper	2.7073051	0.0215567	chr2	sense overlapping	Ola1
mmu_circRNA_19991	hyper	2.6706516	0.0059969	chr1	exonic	Slc9a2
mmu_circRNA_34924	hyper	2.670651559	0.005996879	chr1	exonic	Slc9a2
mmu_circRNA_19150	hypo	3.294895	2.653E-05	chr15	sense overlapping	Arid2
mmu_circRNA_017958	hypo	2.7671474	0.0296858	chr10	exonic	Anks1b
mmu_circRNA_43775	hypo	2.736182	0.0241969	chr9	exonic	Maml2
mmu_circRNA_37703	hypo	2.4422092	0.0010981	chr4	exonic	Ptp4a2
mmu_circRNA_32468	hypo	2.384711	0.0186258	chr19	exonic	Pcgf5
mmu_circRNA_30324	hypo	2.3677878	0.0066817	chr17	exonic	Tsc2
mmu_circRNA_43882	hypo	2.3552045	0.0095023	chr9	sense overlapping	Bmper
mmu_circRNA_28255	hypo	2.3323474	0.0390009	chr15	exonic	Mtmr12
mmu_circRNA_43940	hypo	2.3177426	0.0009251	chr9	exonic	Arhgap32
mmu_circRNA_19555	hypo	2.2504696	1.558E-05	chrX	intronic	XLOC_027457

**Figure 4:**
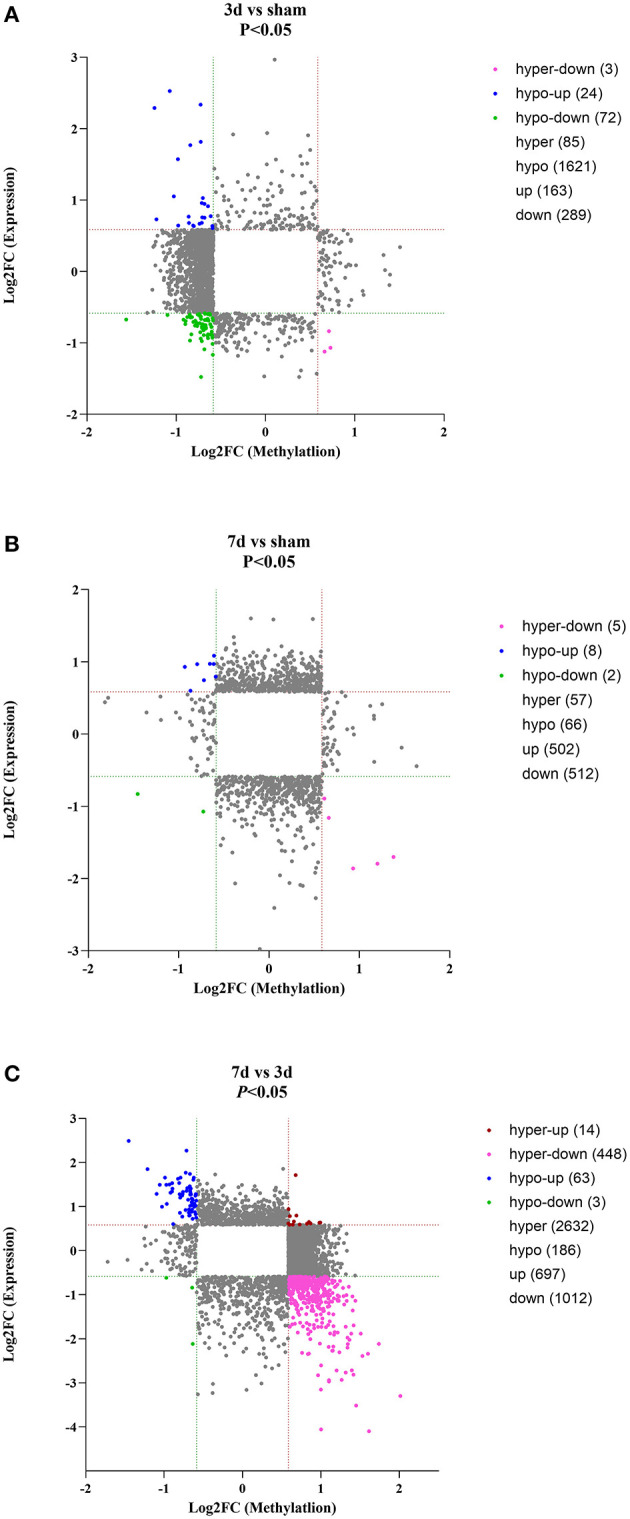
Conjoint analysis for expression and m^6^A methylation profiles of circRNAs around the infarct cortex in 3d vs. sham **(A)**, 7d vs. sham **(B)**, and 7d vs. 3d **(C)**.

As for circRNA expression level, compared with the sham group, numerous circRNAs were differentially expressed, including 163 up-regulated and 289 were down-regulated in the 3d group, while 502 up-regulated and 512 down-regulated in the 7d group. In comparison to the 3d group, 697 circRNAs were significantly increased and 1,012 were decreased in 7 days after MCAO. Besides, we further explored the relation between methylation and expression (Figures 4A–C). We found that in 3d group, 24, 0, 3, and 72 circRNAs were hypomethylated-up expressed, hypermethylated-up expressed, hypermethylated-down expressed, and hypomethylated-down expressed respectively, while turned to 8, 0, 5, 2 ones in 7d group. Compared with the 3d group, there were 63 hypomethylated-up expressed, 14 hypermethylated-up expressed, 448 hypermethylated-down expressed and 3 hypomethylated-down expressed circRNAs in 7d group.

### Verification of the Change in m^6^A Levels of CircRNAs After Cerebral Infarction

Post-stroke alterations of m^6^A modifications demonstrated by m^6^A immunoprecipitation and microarray analysis were confirmed by MeRIP-qPCR. In comparison with the sham group, the methylation levels of mmu_circRNA_27268 and mmu_circRNA_20673 were significantly up-regulated in the 3d group, while mmu_circRNA_32905 was hypomethylated. Although the difference in methylation of mmu_ circRNA_29864 was not statistically significant, there was a downward trend. In general, the validation data was consistent with the m^6^A immunoprecipitation and circRNA microarray analysis (Figure 3G).

### Functional Prediction for Parental Genes of Differentially m^6^A-Modified CircRNAs

**Figure 5:**
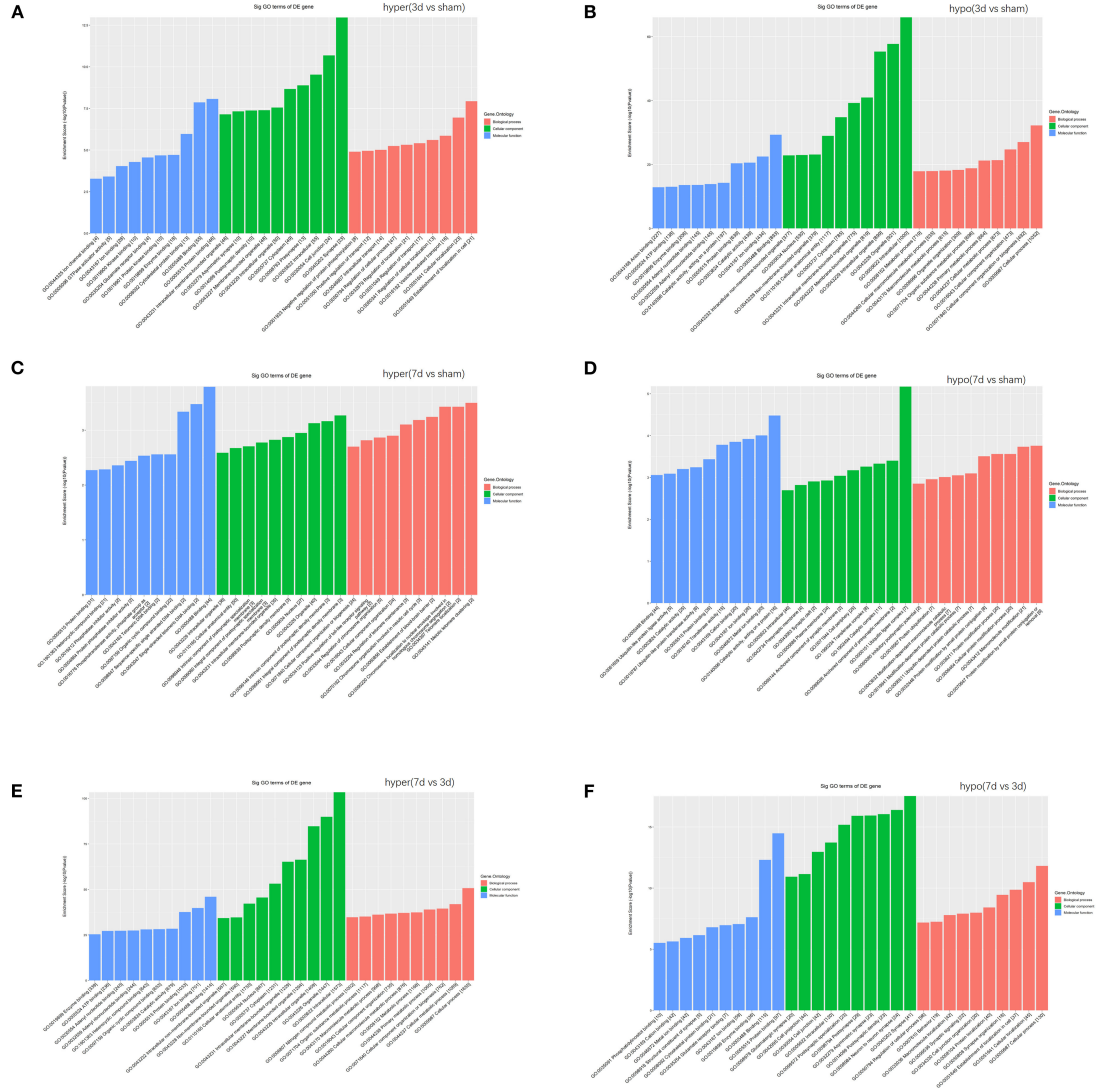
Function analyses of the parent genes of differentially m^6^A-modified circRNAs. Function analyses of the parent genes of significantly hypermethylated and hypomethylated circRNAs around the infarct cortex in 3d vs. sham **(A,B)**, 7d vs. sham **(C,D)**, and 7d vs. 3d **(E,F)**.

To understand the significance of the m^6^A modification in the pathology of cerebral ischemia, GO and KEGG analyses were performed to compare the parental genes of differentially m^6^A-modified circRNAs at 3d or 7d after cerebral infarction. The top 10 most significant enrichment scores in biological processes (BP), cellular components (CC), and molecular functions (MF) are displayed in the histograms (Figures 5A–F). With the development of cerebral ischemia, the BP for hypermethylated circRNAs was established for localization in cells and cellular localization at 3 days after ischemia onset, which displayed a transition to meiotic telomere clustering and establishment of the blood-brain barrier at 7 days after MCAO. Analogously, CC showed enrichment of major synapses and cellular junctions at 3d group, which shifted to integral components of the postsynaptic density membrane at 7d group. We observed the involvement of the glutamate receptor, GTPase activator, single-stranded telomeric DNA binding, and phosphotransferase activity in MF. As for the hypomethylated circRNAs, BP, CC, and MF enrichment concentrated mainly on cellular processes, intracellular, and binding at 3 days after MCAO, which changed to protein ubiquitination, ubiquitin ligase complexes, and ubiquitin-like protein ligase activity at 7 days after MCAO.

### Pathway Prediction for Parental Genes of Differentially m^6^A-Modified CircRNAs

**Figure 6:**
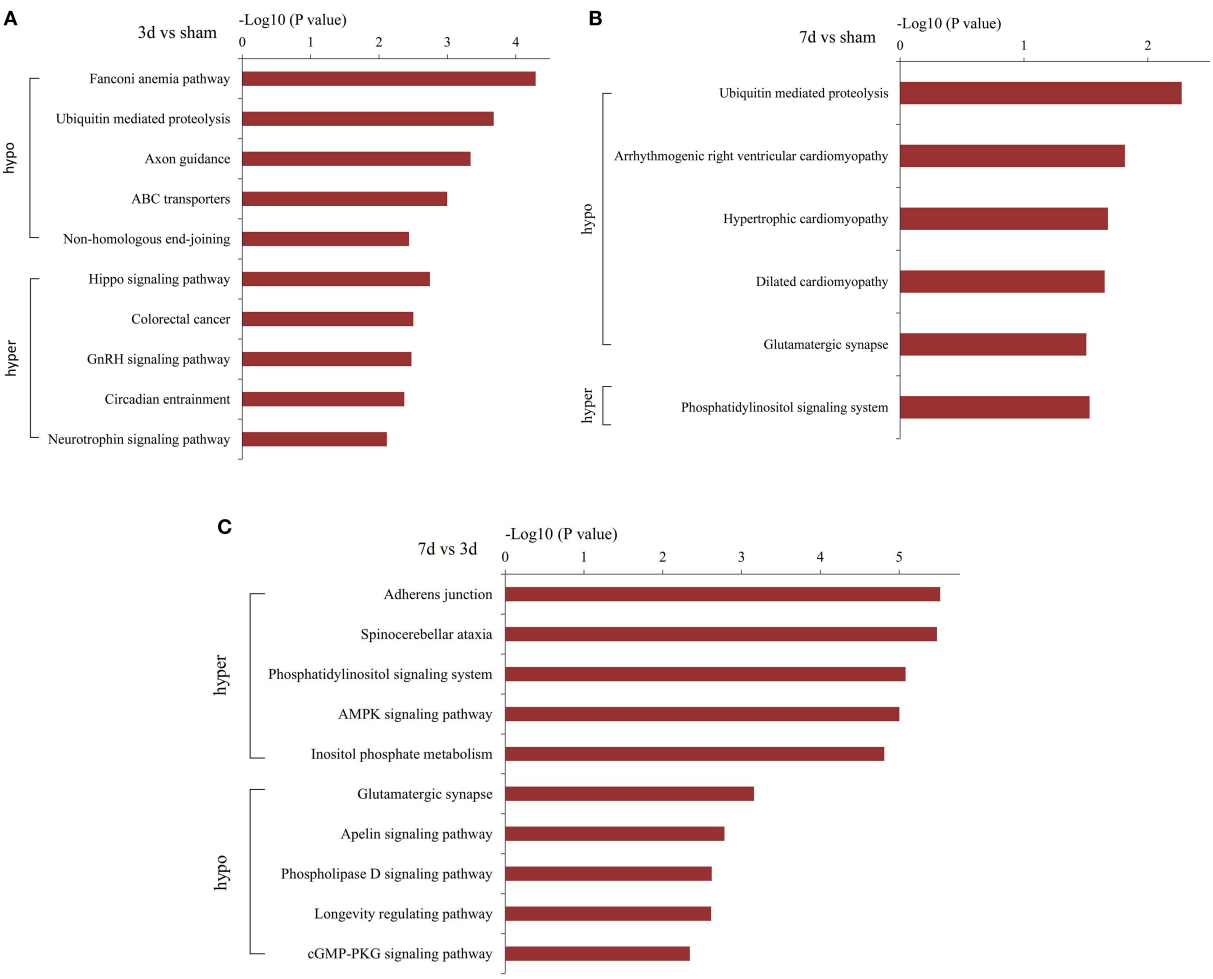
Pathway analyses of the parent genes of differentially m^6^A-modified circRNAs. Pathway analyses of the parent genes of significantly hypermethylated and hypomethylated circRNAs around the infarct cortex in 3d vs. sham **(A)**, 7d vs. sham **(B)**, and 7d vs. 3d **(C)**.

KEGG analysis demonstrated the most enrichment pathways (Figures 6A–C). Compared with the sham group, various pathways including Hippo, neurotrophin, ubiquitin-mediated proteolysis, and axon guidance signaling were the major enrichment pathways at 3 days after MCAO, then turned into phosphorylated inositol signaling, ubiquitin-mediated proteolysis, and the glutamatergic synapse pathway at 7 days after MCAO. As for differentially m^6^A-modified circRNAs between 7d and 3d groups, the pathways mainly enriched in adherens junction, phosphatidylinositol signaling system, AMPK signaling pathway, and glutamatergic synapse.

### The Interaction of CircRNA-miRNA-mRNA

**Figure 7:**
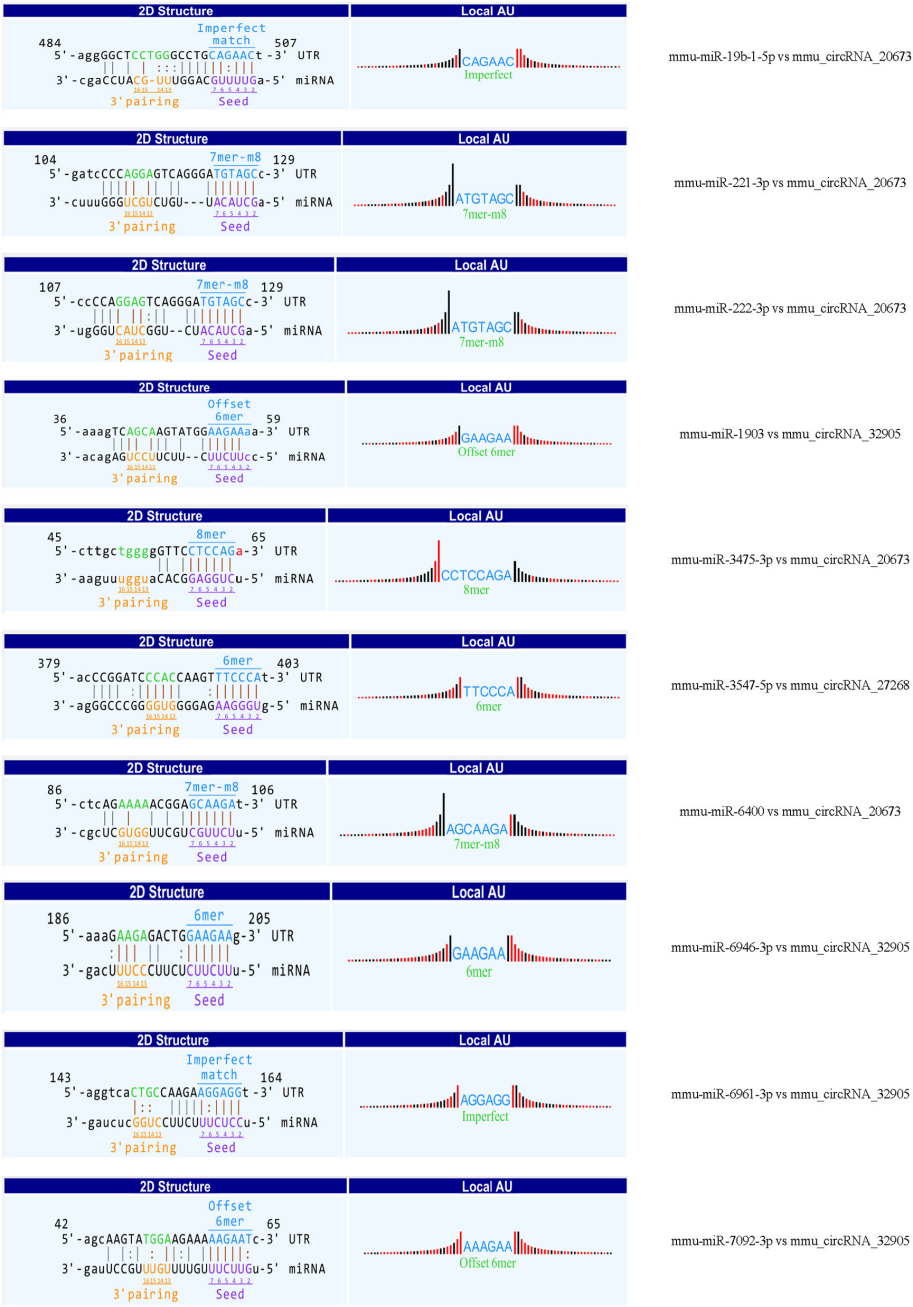
The predicted binding sites between three verified differentially m^6^A-modified circRNAs and ten miRNAs.

**Figure 8:**
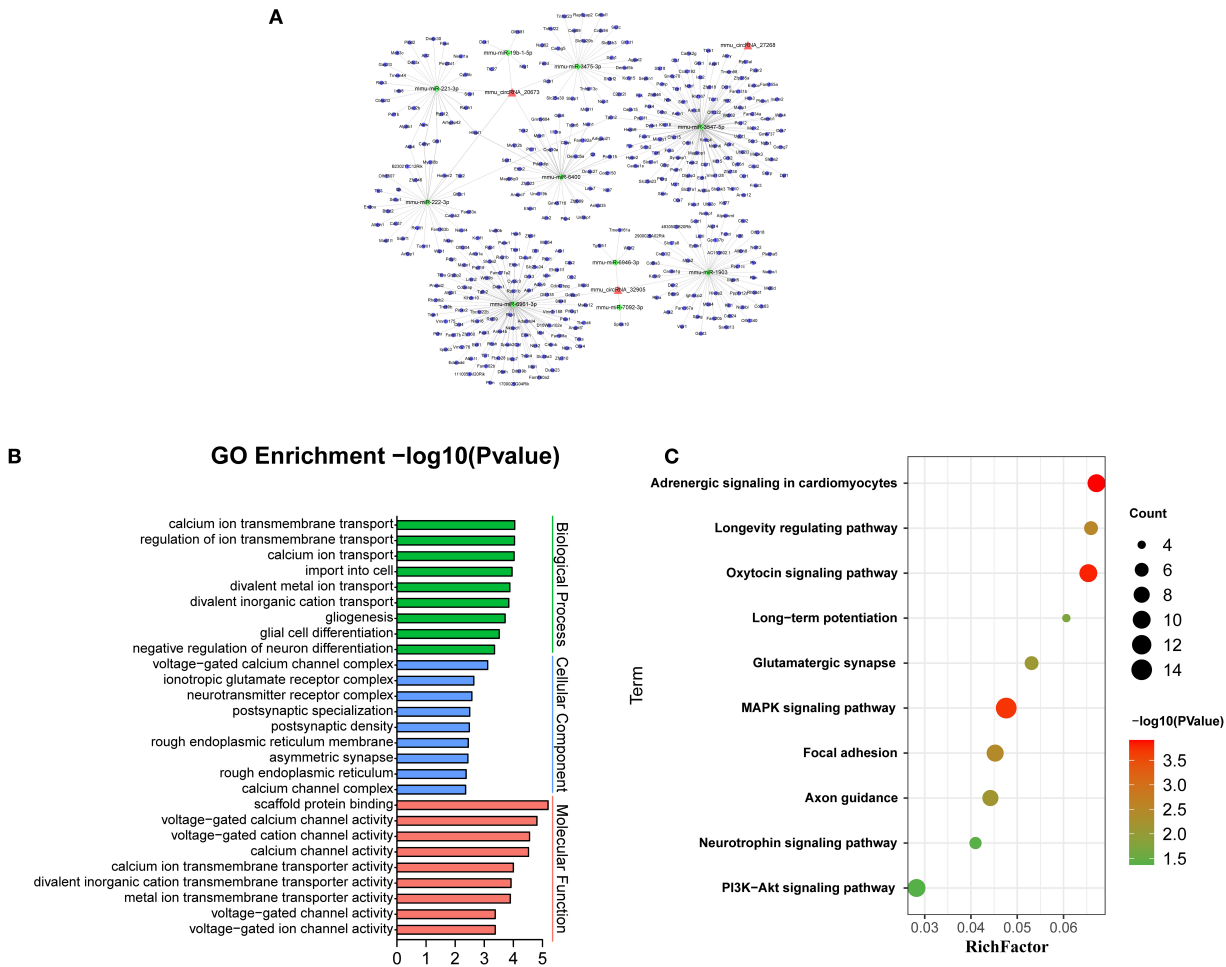
Bioinformatics analysis for target genes of three verified differentially m^6^A-modified circRNAs. **(A)** The circRNA-miRNA-mRNA network contains 3 circRNAs, 10 miRNAs, and 273 mRNAs. **(B)** The top ten most enriched functions of the target genes. **(C)** The top ten most enriched pathways of target genes.

Recent evidences have demonstrated that circular RNAs play a crucial role in regulating the level of miRNA-mediated gene expression by sequestering the miRNAs. Their interaction with disease-associated miRNAs indicates that circular RNAs are important for disease regulation. Ten most significant miRNAs were predicted for three verified m^6^A-circRNAs, and the binding sites were shown in Figure 7. At the same time, we predicted 273 target genes of miRNA and constructed the circRNA-miRNA-mRNA network (Figure 8A). GO analysis indicated that the top ten results of the BP, CC, and MF of target genes were almost related to calcium ion, and the most significant enrichment functions, respectively, were calcium ion transmembrane transport, voltage gated calcium channel complex, and voltage-gated calcium channel activity (Figure 8B). KEGG of target genes revealed the MAPK signaling pathway, focal adhesion, axon guidance, glutamatergic synapse, long-term potentiation, neurotrophin signaling pathway, and PI3K-Akt signaling pathway were mainly enriched pathways (Figure 8C).

## Discussion

It's reported that different pathophysiological processes occur after 3d and 7d of cerebral infarction (F. Zhang et al., 2019), and we took 3d and 7d as time points to explore the dynamic change of m^6^A level after cerebral infarction. Our research suggests that stroke alters the methylation status of circRNAs dynamically. To our knowledge, this is the first study to explore methylation of circRNAs around the cerebral infarct region after MCAO in mice. And we found that the mRNA levels of m^6^A-related enzymes were significantly altered, which were supposed to be a contributing factor of the alteration in methylation of circRNAs. Additionally, we further showed that ALKBH5 and YTHDF3 proteins were changed at different times after stroke. The bioinformatics analysis showed that the differentially methylated circRNAs may act in some pathological molecular events after cerebral ischemia.

Notably, the methylation of circRNAs acts as a regulator of other diseases. It has been reported that decreased m^6^A abundance of methylated circRNAs can be observed in senile cataract and hypoxia-induced pulmonary hypertension (Li et al., 2020; Su et al., 2020), while the abundance is increased in some cancers (He et al., 2021). Therefore, the pathophysiological process of diseases may be related to an imbalance in the N6-methylation of adenosine in circRNAs. Recent studies have shown that stroke significantly changes the global level of m^6^A in peri-infarct tissue, and enzymes associated with the m^6^A modification are believed to mediate post-stroke secondary brain damage by participating in apoptosis and inflammation (Chokkalla et al., 2019; Si et al., 2020; Zhang et al., 2020).

Our results also indicated that the transcripts of these enzymes were differentially expressed between the sham and the MCAO groups, which implies that the homeostasis of m^6^A may change after stroke. Interestingly, the transcription level of the demethylase FTO decreased significantly at 3 days after MCAO compared with the sham group, while it increased significantly at 7d after MCAO compared to the 3d group. This indicates that FTO is decreased in the short term after stroke, which was confirmed by a recent report (Chokkalla et al., 2019). In the brain, FTO and ALKBH5 are predominantly expressed in neurons and increase dynamically during neural development, leading to the proliferation and differentiation of neural stem cells (Li et al., 2017; Chokkalla et al., 2019; Du et al., 2020). Studies have shown the protective role of FTO in proving brain damage and myocardial inflammation by regulating the m^6^A level (Chokkalla et al., 2019; Dubey et al., 2022). Another study suggested that FTO can increase lipid levels, which leads to a decrease in adenosine, an important factor that promotes neuronal apoptosis. Therefore, FTO deficiency can lead to adenosine accumulation, resulting in neuronal apoptosis (Gao et al., 2020). Although ALKBH5 has been reported to selectively demethylate BCL2 transcripts, which can prevent the degradation of BCL2 mRNA, enhance the expression of the anti-apoptotic BCL2 protein, and inhibit neuronal apoptosis (Xu et al., 2020), the role of ALKBH5 is less to know. Our study showed that ALKBH5 protein decreased in 3d group but increased in 7d group, suggesting that it could be one of the potential targets for treatment of stroke, and increases in demethylase may improve cerebral infarction by changing the m^6^A modification. Additionally, YTHDF3 also showed the same trend as ALKBH5, and the latest study showed that YTHDF3 and ALKBH5 were involved in synaptic plasticity (Martinez De La Cruz et al., 2021). However, the specific mechanisms require further study.

Recently, research on m^6^A has focused on the transcription and post-transcriptional processes of mRNAs. Although great progress has been made with mRNAs (Niu et al., 2013), the mechanisms involved in the m^6^A methylation in non-coding RNAs, especially circRNAs, are still poorly understood. Limited research has shown that the m^6^A modification regulates the biogenesis, degradation, stability, translation, and immunogenicity of circRNAs (Chen et al., 2017, 2021; Knuckles and Buhler, 2018; Park et al., 2019; Di Timoteo et al., 2020). CircRNA is produced by selective reverse splicing of primary transcripts, interestingly, the deposition of specific exon regions with m^6^A was suggested to facilitate this process, resulting in the formation of some circRNAs (Di Timoteo et al., 2020). As an m^6^A reading protein, YTHDF2 is known as a degradation tool of mRNAs. YTHDF2 was later found to combine with RNase P/MRP to recognize the degradation of circRNAs with the m^6^A modification (Park et al., 2019). One study showed that circRNAs containing m^6^A motifs were translated in a cap-independent manner, and translation efficiency was related to m^6^A levels levels (Knuckles and Buhler, 2018; Di Timoteo et al., 2020). These pieces of evidence show that the m^6^A modification may act not only on post-transcriptional regulation of mRNAs, but also on circRNAs. Another special regulatory mechanism of m^6^A modification of circRNAs has been demonstrated. Exogenous circRNAs that activate immune responses are thought to be “circFOREIGN”; however, the m6A modification masks them as “circSELF”, which greatly reduces their immunogenicity modifications can directly change the abundance of circRNAs by regulating their biogenesis, degradation, and stability, as well as alter the characteristics of circRNAs, such as their immunogenicity. These studies have demonstrated the potential functions of m^6^A-modified circRNAs in disease regulation.

MeRIP combined with microarray analysis revealed that the m^6^A modification was altered in a considerable number of circRNAs at 3 and 7 days post-cerebral infarction compared with the sham group. In the early stage after cerebral infarction, the majority of circRNAs were hypomethylated, while the circRNAs were hyper-and hypomethylated to same extent in the later stage. Our results indicated that m^6^A methylation was a dynamic process after stroke. Abnormal methylation of circRNAs was supposed to affect multiple molecular events that lead to secondary injury after ischemia. Numerous circRNAs, such as circTTC3, circCCDC9, and circTLK, have been found to regulate autophagy, nerve regeneration, axonal plasticity, and the blood-brain barrier after cerebral infarction (Wu et al., 2019, 2020; Chen et al., 2020; Yang et al., 2020, 2021), and correcting their dysregulation improved the brain damage.

However, the upstream regulatory mechanisms of these circRNAs are not well-known. Interestingly, N6-methylation of adenosine alters the abundance of circRNAs by regulating their generation and degradation (Park et al., 2019; Di Timoteo et al., 2020). Therefore, changing the methylation status may correct the level of circRNAs and affect their functions in cerebral ischemia. Our conjoint analysis of circRNAs' expression and m^6^A methylation profiles screened all circRNAs changed in both methylation and expression levels. We found that the differentially expressed circRNAs mainly shows hypomethylation at 3 days after MCAO. However, the trend of methylation and expression seems to be opposite at 7 days after MCAO. The hypermethylated circRNAs were mainly down-expressed, while the hypomethylated ones were up-expressed. Our study indicated that the methylation level and abundance of circRNAs are not the only relationship. Other research also reported that increasing methylation levels can both increase or decrease the expression of circRNAs (Park et al., 2019; Su et al., 2020; Chen et al., 2021). It will be necessary to investigate the specific mechanisms that alter m^6^A-modified circRNAs in the pathological process after stroke.

CircRNA is derived from the precursor of mRNA. Thus, it can make great significance in pathophysiological process of diseases by regulating its parental gene. To further determine the function of differentially methylated circRNAs around cerebral infarction, we conducted the bioinformatics analysis. Our genomic analysis showed that BP and CC terms were involved in cell localization and intracellular transition at 3 days after MCAO, and shifted to the establishment of the blood-brain barrier and protein ubiquitination at 7 days after MCAO. Moreover, MF term happened the transition of glutamate receptor to phosphotransferase activity. Our results show that the secondary changes after stroke mainly occur from early nerve injury to late brain repair, indicating that differentially methylated circRNAs may regulate these pathological processes. In addition, previous research displayed that cerebral ischemia caused oxidative stress, neurovascular regeneration, and synaptic formation (Mehta et al., 2007). In our study, numerous signaling pathways, including ubiquitin-mediated proteolysis, axon guidance, hippo, neurotrophin, glutamatergic synapse, adherens junction, and phosphatidylinositol signaling, were significantly enriched. These pathways regulate the processes of oxidative stress, nerve regeneration, angiogenesis, synaptic formation, and plasticity after cerebral ischemia (Reichardt, 2006; Ding et al., 2014; Zhao et al., 2016). Glutamate is an excitatory neurotransmitter that mediates excitatory synaptic transmission. During cerebral ischemia and hypoxia, glutamate is released in large quantities, which over-activates glutamate receptors, leads to excessive Ca^2+^ influx, and causes brain damage and neuronal death (Mayor and Tymianski, 2018), a major cause of secondary brain injury. Therefore, understanding the regulation mechanism of m^6^A-modified circRNAs in the glutamate signaling pathway might be helpful for better intervention and prognosis and reduces secondary brain injury after stroke.

CircRNA can act as a sponge of miRNA to reduce the inhibitory effect of miRNA on mRNA to promote the expression of mRNA (Wu et al., 2019). Therefore, we selected the verified circRNAs to predict the network of circRNA-miRNA-mRNA. BP, CC, and MF show the predicted mRNAs mainly involve calcium ion transmembrane transport, voltage-gated calcium channel complex, voltage-gated calcium channel activity. Research shows calcium overload occurs in damaged neurons to mediate mitochondrial swelling injury and neuronal necrosis in the peri-ischemic cortex (Pivovarova and Andrews, 2010). It is reported that target genes cacna1g, tpcn2, panx1, and Fyn are involved in pathophysiological processes such as neuroexcitatory conduction, autophagy, inflammation, and synaptic plasticity in cerebral infarction (Shin et al., 2008; Shestopalov and Slepak, 2014; Guo et al., 2020; Rajani et al., 2021). In addition, 10 miRNAs have been predicted in our circRNA-miRNA-mRNA network. At present, little research has focused on these miRNAs. Interestingly, mmu-mir-3547-5 has been reported to play an important role in anti-atherosclerosis, and atherosclerosis is related to the pathogenesis of cerebral infarction (Zhang et al., 2018). In this network, mir-3547-5p and tpcn2 were the downstream targets of mmu_circRNA 27268 and mmu-mir-3547-5p, respectively. Therefore, we speculated that the mmu_circRNA 27268, mir-3547-5p, and tpcn2 may form a network axis, and participate in the pathological process of cerebral infarction by regulating calcium overload.

## Conclusion

In summary, this study is the first to show changes of m^6^A methylation in circRNAs in cerebral infarction. The bioinformatics analysis predicted the possible functions and related pathways of m^6^A modified-circRNAs in secondary injury and the repair of cerebral infarction. However, understanding how the methylated circRNAs regulate the post-stroke pathophysiological mechanism will require further investigation. Our results provide new insights into the molecular mechanism of ischemia stroke.

## Data Availability Statement

The datasets presented in this study can be found in online repositories. The names of the repository/repositories and accession number(s) can be found below: https://www.ncbi.nlm.nih.gov/geo/query/acc.cgi?acc=GSE196448.

## Ethics Statement

The animal study was reviewed and approved by Institutional Animal Use Committee of the Shenzhen Hospital of Southern Medical University.

## Author Contributions

FL and LL conceived and designed the study and interpreted experiments. YL performed the experiments and wrote the manuscript. HL performed the experiments. YL, XL, ZC, and WZ analyzed the data. All authors read and approved the final submission.

## Funding

This study was supported by the grants from the Science and Technology Project of Shenzhen (JCYJ20190814112201888), Sanming Project of Medicine in Shenzhen (SZSM201812047), Shenzhen Key Medical Discipline Construction Fund (SZXK074), and Guangdong Basic and Applied Basic Research Foundation (2018A030313586).

## Conflict of Interest

The authors declare that the research was conducted in the absence of any commercial or financial relationships that could be construed as a potential conflict of interest.

## Publisher's Note

All claims expressed in this article are solely those of the authors and do not necessarily represent those of their affiliated organizations, or those of the publisher, the editors and the reviewers. Any product that may be evaluated in this article, or claim that may be made by its manufacturer, is not guaranteed or endorsed by the publisher.
